# A Leucine-Rich Receptor Like Kinase LRK2 Is Involved in the Regulation of Cold Tolerance and Yield in Rice

**DOI:** 10.3390/plants13243569

**Published:** 2024-12-21

**Authors:** Huan Yi, Yarui Zhao, Xinting Wu, Mengting Zhang, Minwei Pan, Siyuan Ding, Jiaqi Huang, Qing Gu, Xiaojun Zha

**Affiliations:** College of Life Sciences, Zhejiang Normal University, 688 Yingbin Road, Jinhua 321004, China; yh1262471391@163.com (H.Y.); zhaoyarui@163.com (Y.Z.); 19817952055@163.com (X.W.); 18271376659@163.com (M.Z.); 15088257685@163.com (M.P.); 15925845509@163.com (S.D.); 13728485857@163.com (J.H.); gunc1204@foxmail.com (Q.G.)

**Keywords:** cold stress, *LRK2*, rice receptor-like protein, yield

## Abstract

Low temperature affects rice growth and yield. Receptor-like protein kinases play an important role in plant growth and development. In order to reveal the role of a leucine-rich receptor like kinase LRK2 in low temperature stress and growth and development of rice. In this study, we used the obtained *LRK2* overexpressing plants for experiments and the results show that the cold tolerance and yield of *LRK2* overexpressing plants were higher than that of wild type. *LRK2* has high homology with the phytosulfokine receptors (PSKRs) gene of different species, and *LRK2* gene is responsive to phytosulfokine (PSK). In addition, we observed that the proline content of *LRK2* overexpressing plants was significantly higher than that of the wild-type at low temperature, while the malondialdehyde content was significantly lower than that of the wild-type. Yeast two-hybrid screening and bimolecular fluorescence complementary analysis showed that LRK2 interacts with rice growth hormone response factor 24 (OsARF24) in vitro. These results suggest that *LRK2* gene may be involved in the regulation of cold stress response and yield in rice. These findings will help us understand PSKR signaling in other grasses and support improvements in rice genetics.

## 1. Introduction

Rice (*Oryza sativa*) plays an important role in global food production, and is a major food source for more than one-third of the world’s population [1]. Tillering is one of the most important factors affecting rice yield. Rice is sensitive to low-temperature stress [2,3], which affects rice growth and yield. Furthermore, low temperature is one of the key factors limiting the global distribution of rice. In order to improve the cold tolerance of plants, researchers solved this problem by increasing the expression of genes related to cold stress. For example, cold stress response gene *COLD1* encodes a regulator of G-protein signaling that localizes to the plasma membrane and endoplasmic reticulum to enhance cold tolerance [4]. Rice mitogen-activated protein kinase 3 (OsMAPK3) phosphorylates rice basic HELIX-LOOP-HELIX 2 (OsbHLH002)/interactor of little elongation complex ELL subunit 2 (OsICE1) to enhance rice cold tolerance [5]. NAC domain transcription factor (OsNAC5) can interact with bZIP transcription factor (OsABI5), enhance the stability of OsABI5 protein, and then regulate the expression of downstream cold response (*CORs*) gene, and finally realize the rapid and accurate response of rice to cold stress [6]. The receptor-like kinase (RLK) calcium/calmodulin-regulated receptor-like kinase 1 (CRLK1) can regulate the activity of MAPK by activating the expression of plant cold-inducible genes (COR), making plants tolerant to cold stress [7].

Leucine rich repeat (LRR)-RLKs are the largest family of RLKs in plants [8], which play a role in many plant processes, including members involved in plant growth and development [9,10], and those involved in abiotic and biotic stresses [11,12]. The LRR-RLKs contain three functional domains: the extracellular structural domain that receives signals, the transmembrane structural domain, and the intracellular kinase domain that transduces signals to specific downstream substrates [13,14]. Peptide signaling is an integral part of cell-to-cell communication that helps to relay the information responsible for coordinating cell proliferation and differentiation. The phytosulfokine receptor (PSKR) is a transmembrane LRR-RLK family protein with a binding site for the small signaling peptide, phytosulfokine (PSK). PSK signaling through PSKR promotes normal growth and development, and also plays a role in defense responses. Like other RLKs, these PSKRs might have a role in signal transduction pathways related to abiotic stress responses. Rice phytosulfokine receptors 15 (OsPSKR15), a phytosulfokine receptor from rice, enhanced the abscisic acid response and drought stress tolerance in *Arabidopsis thaliana* [15]. Phytosulfokine receptors 1 (PSKR1) plays a positive role in defense response against *Bacillus cinerea* in tomato [16]. In *Arabidopsis thaliana*, *PSKR1* overexpressing plants had enhanced hypocotyl and root growth [17,18]; and in rice, *PSKR1* overexpressing plants enhanced plant defense against rice bacterial streak disease. However, PSK has not been reported to be associated with cold stress.

*LRK2* is a gene located on chromosome 2, which belongs to the same gene family as *LRK1*-*LRK8* [19]. In previous studies, it was found that *LRK1* can improve rice yield by increasing the number of effective tillers of rice [20], and *LRK2* overexpressing plants in Nipponbare could improve the drought tolerance of rice [21]. In this study, the cloned *LRK2* overexpression vector was transferred into *Oryza sativa L. ssp. indica var.* Zhong hua11 to explore the function of *LRK2*, and the results showed that *LRK2* overexpressing plants had higher tiller number and thousand-grain weight than the wild-type, and the cold tolerance was stronger than the wild-type, suggesting that the *LRK2* gene may play a role in rice growth and development as well as in cold stress response.

## 2. Results and Analysis

### 2.1. Features of LRK2

In order to further explore the relationship between LRK2 and PSKR, we searched the *PSKR1* (AtPSKR1: At2g02220) and *PSKR2* (AtPSKR2: At5g53890) genes of *Arabidopsis thaliana*, *Daucus carota PSKR1* (DcPSKR1: BAC00995.1) gene and *Solanum lycopersicum PSKR1* (SlPSKR1: LOC101246169) gene on NCBI, and compared the sequences of these genes with *LRK2* (OsLRK2: LOC4328340). The results showed that the similarity between *LRK2* and these genes was more than 96% (Supplementary Material: Figure S1A). In addition, we used GenDoc 2.7 software to compare the highly conserved protein structural regions of these genes (Supplementary Material: Figure S1B). Through the above sequence comparison results, we hypothesized that the *LRK2* is related to PSK. Therefore, we cultured 14-day-old rice seedlings with 1 μmol/L PSK-α solution to determine the ex-pression level of *LRK2* gene at different time points. The results showed that the expression level of *LRK2* was significantly up-regulated after PSK treatment, reaching a higher level on day 2 (Supplementary Material: Figure S1C). These results suggest that *LRK2* may have the same function as *PSKRs*.

### 2.2. Expression of LRK2 in Transgenic Rice

**Figure 1:**
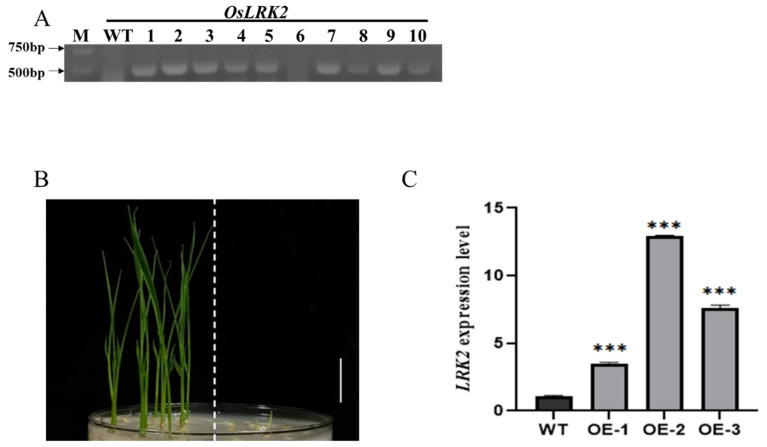
Expression of *LRK2* in transgenic rice. (**A**) Transgenic identification of *LRK2.* (**B**) Chaotropic medium screening of transgene-positive homozygous plants (**left**: transgenic plants, **right**: wild-type plants, bar = 2 cm). (**C**) *LRK2* gene specific primers were used to analyze the relative expression levels of *LRK2* in wide-type plants and *LRK2* overexpressing plants by RT-qPCR (*t*-test, “***” is *p* < 0.001).

The whole *LRK2* gene expression vector was transferred into the wild-type plants (*Oryza sativa L. ssp.* japonica cv. Zhonghua11) by Agrobacterium-mediated transgene technology, and *LRK2* T0-generation plants were obtained. Subsequently, DNA was extracted from the leaves of the seedlings, and the transformation success was identified with the specific primers of the vector hummycin B phosphotransferase gene using pCAMBIA1300s. The wild-type was used as a negative control (Figure 1A). The electrophoretic band amplification of most seedlings was about 400 bp compared to the wild-type plants. According to Mendelian inheritance laws, plants can obtain stable genetic offspring in T2 generation, so we selected *LRK2* positive plants and wild-type lines in T2 generation, and screened stable genetically homozygous plants on 1/2 MS medium containing humomycin resistance. Homozygous plants with hygromycin B resistance grew normally in medium, while wild-type plants were stunted (Figure 1B). Three T2-generation *LRK2* homozygous lines were selected by this method. Real-time fluorescence quantitative PCR was used to detect the expression of *LRK2* in the three homozygous lines and wild-type plants. The results showed that *LRK2* was strongly expressed in these three homozygous lines, and the expression level was significantly higher than that of the wild-type (Figure 1C), indicating that these three homozygous lines were *LRK2* overexpressing homozygous lines (OE-1, OE-2, OE-3). This laid a foundation for further study on the function of *LRK2* overexpressing plants.

### 2.3. Effects of LRK2 Overexpression on the Agronomic Traits of Rice

**Figure 2:**
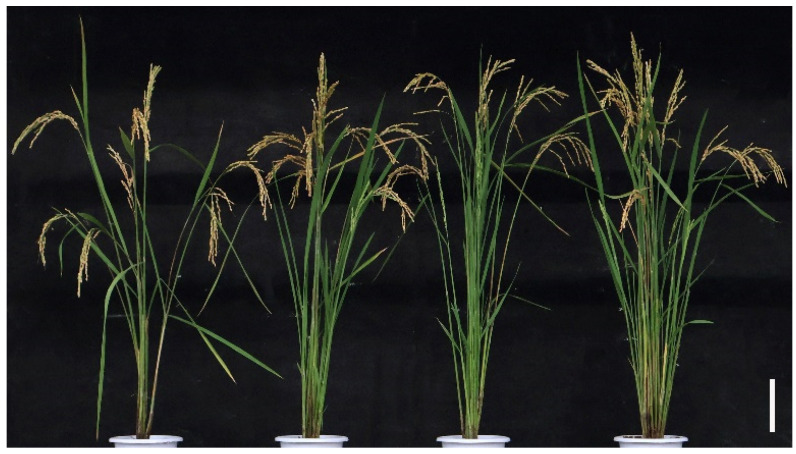
At maturity, the phenotypes of wild-type and *LRK2* overexpressing lines, from left to right, are wild-type: WT, *LRK2* overexpressing lines: OE-1, OE-2, OE-2. Bar = 1 cm.

In order to study the effect of *LRK2* overexpression on rice yield, three *LRK2* overexpressing lines and wild-type plants were planted in the field. At the mature stage of rice, through observing the phenotypes of wild-type and *LRK2* overexpressing lines, it was found that the plant height of *LRK2* overexpressing lines was higher than that of wild-type, and the number of tillers was higher than that of wild-type (Figure 2). In addition, the number of tillers, plant height, 1000-grain weight, primary shoot, secondary shoot, ear length and grain number per spike of wild-type and *LRK2* overexpressing lines OE-1, OE-2 and OE-3 were statistically analyzed, and each plant was systematically measured at 10 plants. According to Table 1, the number of tillers, thousand grain weight, spike length and number of grains per spike of *LRK2* overexpressing plants were significantly higher than those of wild-type. In conclusion, *LRK2* overexpressing plants can increase rice yield by increasing tillering number, 1000-grain weight and grain number per panicle.

### 2.4. Overexpression of LRK2 Improved the Tolerance of Rice to Low Temperature Stress

**Figure 3:**
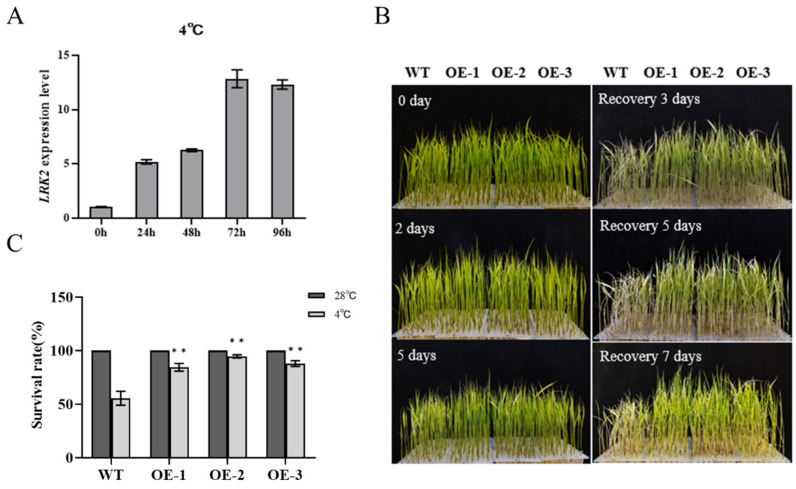
Phenotypes of *LRK2* overexpressing plants under low temperature (4 °C) stress. (**A**) Expression of the *LRK2* gene in wide-type plants subjected to 4 days of low-temperature treatment. (**B**) 0 days: wide-type plants and *LRK2* overexpressing (OE) plants were grown for 14 days under normal conditions. 2, 5 days: wide-type plants, *LRK2* overexpressing plants treated with 4 °C low temperature for 2 and 5 days. Recovery 3, 5, 7 days: wide-type plants and *LRK2* overexpressing plants were transferred to normal conditions after low-temperature treatment for 3, 5, and 7 days. (**C**) Survival rate of wide-type plants and *LRK2* overexpressing plants after recovery under normal conditions. “**” is *p* < 0.01.

To investigate the mechanism of the *LRK2* response to abiotic stresses, we examined the expression level of *LRK2* after low temperature (4 °C) treatment. The results showed that the expression of the *LRK2* gene increased after low temperature treatment, reaching a maximum level at 72 h (Figure 3A). Consequently, we speculated that *LRK2* might be involved in low-temperature stress. Therefore, we examined the performance of wild-type plants and *LRK2* overexpressing lines under low-temperature stress (Figure 3B). Under normal growth conditions, wild-type plants and *LRK2* overexpressing plants grew well. At the four-leaf stage, the rice seedlings were placed in a low-temperature environment. After two days of treatment, no significant changes in the growth of wide-type plants and *LRK2* overexpressing lines OE-1, OE-2, and OE-3 were found. After five days of cold treatment, yellowing and curling of leaves were found in both the wild-type and *LRK2* overexpressing lines OE-1, OE-2, and OE-3. After the cold stress treatment, the plants were allowed to recover by placing them in an incubator under normal conditions, and the growth of the plants was observed. Compared with that of the wild-type plants, the recovery and growth of the *LRK2* overexpressing lines OE-1, OE-2, and OE-3 were more vigorous. When the survival rate of plants was determined on the seventh day of recovery, the survival rate of the wild-type was 55.6%, whereas the survival rates of OE-1, OE-2, and OE-3 were 84.5%, 94.6%, and 87.9%, respectively (Figure 3C). The above result indicated that *LRK2* overexpressing plants regulates the cold stress mechanism in rice.

**Figure 4:**
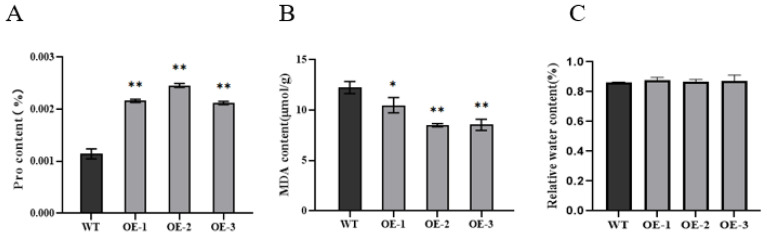
Physiological data of wild-type (WT)and *LRK2* overexpressing plants (OE-1, OE-2, OE-3) growing to 14 days of age were analyzed after 5 days of treatment under low temperature stress (4 °C). (**A**) Proline (Pro) content of wide-type plants and *LRK2* overexpressing plants. (**B**) Malondialdehyde (MDA) content of wide-type plants and *LRK2* overexpressing plants. (**C**) Relative water content. “*” is *p* < 0.05, “**” is *p* < 0.01.

In order to further prove that *LRK2* overexpression regulates cold stress in rice, we determined the contents of proline and malondialdehyde and relative water content of wild-type plants and OE-1, OE-2 and OE-3 plants were determined after 5 days of cold stress treatment. The results showed that the proline content of *LRK2* overexpressing plants was significantly higher than that of wild-type plants (Figure 4A). The content of malondialdehyde in *LRK2* overexpressing plants was significantly lower than that of wild-type plants (Figure 4B). There was no significant difference in relative water content between wild-type and *LRK2* overexpressing plants (Figure 4C). *LRK2* overexpressing plants are involved in the regulation of cold tolerance of rice.

### 2.5. Interaction Between LRK2 and Auxin Response Factor OsARF24

**Figure 5:**
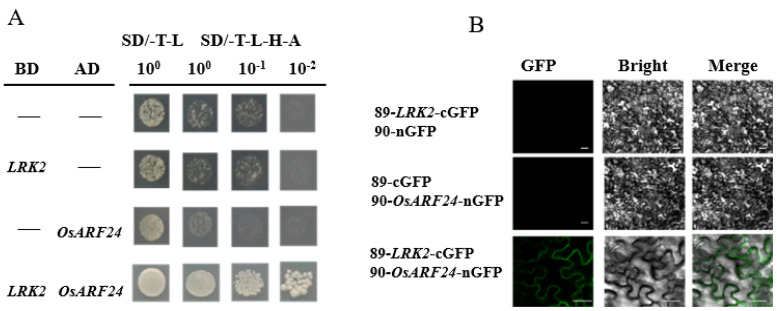
In vitro interaction of LRK2 with OsARF24. (**A**) LRK2 interacts with OsARF24 in SD/-Trp-Leu (T−L) medium and SD/−Trp−Leu−His−Ade (T−L−H−A) medium. (**B**) Verification of the interaction between LRK2 and OsARF24 by BiFC was observed by transfecting the constructed vector into tobacco. Scale bar = 50 nm. LRK2, leaf rust kinase-like 2; OsARF24, auxin response factor 24; BiFC, bimolecular fluorescence complementation; GFP, green fluorescent protein; AD:OsARF24−pPR3−N, BD:LRK2−PBT3−SUC.

Auxin plays an important role in plant growth and development. This study found that *LRK2* overexpressing plants showed increased tillering and increased plant height. In order to explore whether the growth and development of *LRK2* overexpressing plants were related to auxin. In this study, a growth hormone response factor OsARF24 interacting with LRK2 was screened through yeast two-hybrid experiment (Figure 5A). To further verify the interaction between the two proteins, we performed the BiFC experiment. Cells carrying LRK2:cGFP and OsARF24:nGFP fluoresced green under 395 nm light, reliably confirming the interaction between LRK2 and OsARF24 (Figure 5B). This experiment lays a foundation for further exploration of the molecular mechanism of LRK2 and OsARF24.

## 3. Discussion

Cold stress is an important environmental factor affecting crop yield. Low temperature stress in plants causes metabolic disorders and affects their growth and development.

In this study, *LRK2* overexpressing plants were obtained, which confirmed the role of *LRK2* overexpressing plants in rice growth and development and response to low temperature stress. The results showed that *LRK2* overexpressing plants had increased yield due to having higher tillering number and 1000-grain weight (Table 1). Under low temperature stress, the expression level of *LRK2* gradually increased with time (Figure 3A). The cold tolerance of *LRK2* overexpressing seedlings was higher than that of the wild-type, and the survival rate after low temperature treatment was as high as 94.6%, while that of the wild-type was only 55.6% (Figure 3B). These results suggest that *LRK2* plays an important role in rice growth and development and low temperature stress. In addition to that, under low temperature stress, plants will enhance their osmoregulation ability by changing the content of various osmoregulatory substances, so as to improve the tolerance of plants to low temperature environment. These osmoregulatory substances include proline, some soluble sugars and proteins. For example, proline (Pro) can be used as a physiological indicator to judge plant tolerance to low temperature stress. The content of proline under normal conditions is not high, but its content can be significantly induced by low temperature [22], and the higher the content of proline in plants, the higher the content of proline in plants. The tolerance of plants to low temperature stress is also higher [23,24]. In addition, low temperature stress will destroy the balance of oxidative metabolism in cells, cause continuous accumulation of reactive oxygen species (ROS), damage the cell membrane system of plants, and also lead to the conversion of polyunsaturated fatty acids into malondialdehyde (MDA), thus causing damage to plants [25]. Therefore, the degree of cell membrane oxidative damage can be identified by the content of malondialdehyde [26], and the content of malondialdehyde is also an important reference for the low temperature tolerance of plants. We determined malondialdehyde, proline and relative water content in wild-type and *LRK2* overexpressing plants under low temperature stress, and found that the content of malondialdehyde in *LRK2* overexpressing lines was lower than that of wild-type, and the content of proline was higher than that of wild-type, and there was no significant difference in relative water content (Figure 4). These results suggest that *LRK2* overexpressing plants can improve the cold tolerance of rice by regulating the content of malondialdehyde and proline in vivo.

LRK is a receptor on the surface of plant cells that can sense external signals and transmit them to the cell, triggering downstream reactions through phosphorylation. PSKR, as an exopeptide receptor, binds specifically to PSK, thereby promoting the interaction of PSKR with kinases, cyclic nucleotide-gated channel proteins, and calmodulin. This regulates plant growth and development, and the stress defense response via phosphorylation, cGMP production, and Ca^2+^ inflow [27]. A comparison of their protein structures showed that LRK2 and PSKR have a high homology, and it was speculated that *LRK2* might be functionally similar to *PSKR* (Supplementary Material: Figure S1A). By soaking seedlings in PSK-α and measuring the expression level of *LRK2* gene in the seedlings, we found that *LRK2* expression increased significantly, indicating that *LRK2* responds to PSK- α(Supplementary Material: Figure S1C). Therefore, we speculate that there may be some regulatory relationship between *LRK2* and PSK, which needs to be verified by follow-up experiments.

As a class of plant-specific transcription factors, auxin response factors (ARFs) specifically bind to some auxin response elements to regulate the expression of auxin response genes [28]. With the continuous improvement of genome sequencing technology, the ARF gene family has been detected in a variety of crops, including food crops such as wheat, rice, and potato, as well as cash crops, such as tomato, peach and citrus [29]. Auxin response factors proteins (ARFs) play an important role in regulating the growth and development of plants. Currently, the ARF gene family is known to play important roles in organ development and vegetative growth [30], the regulation of lateral root formation [31], anther development, and pollen formation [32], often showing species expression specificity. At the same time, *ARF* transgenic plant show complex and diverse forms under abiotic stresses. The results showed that the expression level of *ARF* gene in licorice and eggplant under high salt stress was higher than that before treatment [33,34], and the stagnant flooding tolerance of lowland rice is directly related to its ARF genes [35]. In this study, we found that LRK2 interacts with OsARF24 through yeast two-hybrid experiment and bimolecular fluorescence complementary experiment (Figure 5). Therefore, we speculated that *LRK2* can promote the growth and development of rice and regulate its cold tolerance through interaction with *OsARF24*.

Because overexpressing plants have certain limitations in verifying plant function. According to the above results, we can only preliminarily determine that the leucine-rich receptor like kinase LRK2 is involved in the regulation of cold tolerance and yield in rice. To accurately confirm that *LRK2* positively regulates cold tolerance in rice, *LRK2* mutant materials need to be obtained later for experimental verification. In addition, more experiments are needed to verify the regulatory relationship between PSK, *OsARF24* and *LRK2*.

## 4. Experimental Procedure

### 4.1. PSK Processing

Seedlings grown to two weeks old were transferred to a solution containing 1 μmol/L of PSK-α for further culture. The samples were collected before culture and at 1, 2, 4, 6, and 8 days after culture, and were quickly frozen in liquid nitrogen to extract RNA, which was reverse transcribed into cDNA. RT-qPCR was then performed to determine the change in *LRK2* expression level.

### 4.2. Generation of Transgenic Rice

The full-length cDNA sequence of the *LRK2* gene was amplified from wild-type plants, and the fragment was inserted into vector *pCAMBIA 1300-2×35S* under the control of the 35S promoter from cauliflower Mosaic virus to construct the *LRK2* overexpression vector. The constructed vector was transferred into wide-type plants using *Agrobacterium*-based transformation, and the positive transgenic plants were identified using PCR with hygromycin universal primers.

### 4.3. RNA Isolation and RT-qPCR Analysis

An RNA extraction kit was used to extract total RNA (Tiangen, Beijing, China), and an RT-PCR reverse transcription kit was used to reverse transcribe the RNA into cDNA (CWBIO, Taizhou, China), both according to the manufacturer’s instructions. The diluted product was used as the template for RT-qPCR analysis. The primers used for RT-qPCR analysis were 5′-TGCCTGCAAGCCACATATCA-3′ and 5′-AGCTCAAGCAGGACAACTCC-3′, and the relative gene expression was calculated using the Actin gene as the internal reference and the 2^−ΔΔCT^ method [36]. Each data point represented three replicates, and each experiment was repeated three times.

### 4.4. Growth Conditions

In order to promote seed germination, wild-type rice seeds and transgenic LRK2 rice seeds were soaked in water, wrapped in wet cotton cloth, and placed in an incubator at 37 °C. After germination, the seeds were cultured for 14 days in an incubator with light at 28 ° C, dark at 26 ° C, 12 h and 65% humidity. The cold stress experiment of rice was carried out with 14-day-old seedlings

### 4.5. Cold Stress Treatment

Fourteen-day-old *LRK2* overexpressing plants and wild-type plants were cultured at 4 °C. After the wild-type plants showed the leaf curl phenomenon, the recovery treatment began. The *LRK2* overexpressing plants and wild-type plants were placed under normal conditions for growth and culture, during which the phenotypes of the plants were observed and photographed. After recovery treatment, the survival rate statistics were determined. Generally speaking, plants with fresh green leaves were viable plants.

### 4.6. Determination of the Proline and Malondialdehyde Contents

Leaf tissues from wild-type plants and *LRK2* transgene plants subjected to abiotic stress were cut into small segments, ground in quartz sand, and then extracted with corresponding extraction reagents. After extraction, enzyme markers were used for proline and malondialdehyde content determination, as described previously [37]. Each data represented three replicates, and each experiment was repeated three times.

### 4.7. Yeast Two-Hybrid Analysis

LRK2 was used as the bait protein and its coding region was fused with vector PBT3-SUC to construct the bait vector. OsARF24 was used as the prey protein and its coding region was fused with vector pPR3-N vector to construct the prey vector. PBT3-SUC, pPR3-N, LRK2-PBT3-SUC, and OsARF24-pPR3-N were transferred into yeast strain NMY51, which was inoculated on SD/ -Leu-Trp and SD-Trp -His-Leu-Ade medium, and incubated at 28 °C for 3 days. The primers used included LRK2-PBT3-SUC-F (5′-GGCCATTACGGCCATGCAGCCACCT-3′) and LRK2*-*PBT3-SUC-R (5′-GGCCGAGGTGGCCATGTCGGAGCCT-3′), OsARF24-pPR3-N-F (5′-GATTACGCTGGATCCATGGCTGCTGCAGCGACGGC-3′), and OsARF24-pPR3-N-R (5′-TGACTCGAGGTCGACGGGCAGTTCTCAGAATTCAGTG-3′).

### 4.8. Bimolecular Fluorescence Complementation Assay

Primers 5′-GCAGGCTCCGAATTCATGCTTTTCTCGCTCAGG-3′ and 5′-AAGCTGGGTCGAATTCGTCGGAGCCTACACT-3′ were used to amplify amplification the *LRK2* cDNA fragments, and primers 5′-GCAGGCTCCGAATTCATGGCTGCTGCAGCG-3′ and 5′-AAGCTGGGTCGAATTCGCAGTTCTCAGAATT-3′ were used to amplify *OsARF24* cDNA fragments. The amplified cDNAs were cloned into a vector expressing green fluorescent protein (GFP) with fluorescent tags at the N and C ends, respectively [38]. The constructed vectors were sequenced for verification and the correct vectors were transformed into *Agrobacterium*. Then, *Agrobacterium* with *LRK2*:cGFP and *OsARF24*:nGFP were mixed with RNA Silencing Suppressor p19 in equal proportions and centrifuged. The suspension was injected into the leaves of 3-week-old tobacco plants. The injected tobacco plants were incubated in the dark for 24 h and then cultured in the normal culture environment for 2 days. Finally, the tobacco leaves were observed under a confocal microscope.

### 4.9. Phylogenetic Analysis

Phylogenetic analysis of LRK2 and other PSKR was carried out using MEGA 6.0 [39] and GenDoc2.7.

## 5. Conclusions

**Figure 6:**
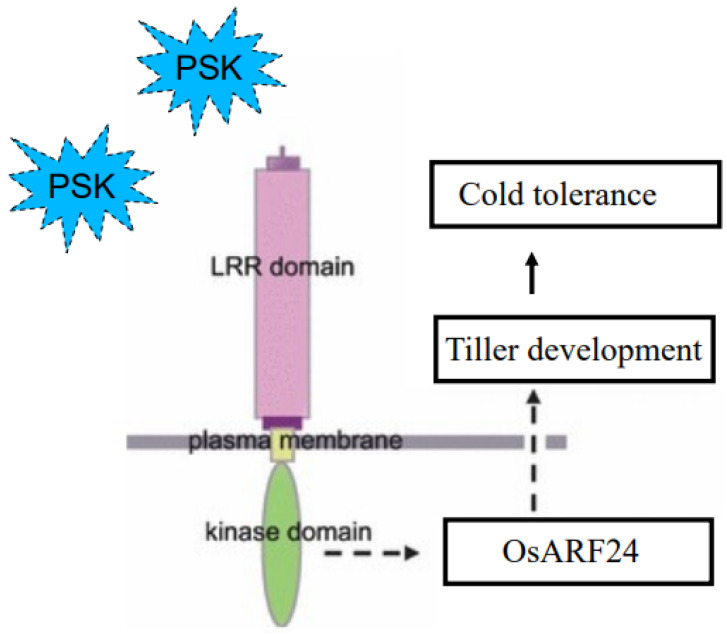
Schematic diagram of *LRK2* improving rice yield and cold resistance. The dotted boxes and lines in the figure represent a hypothesis.

In summary, the results of this study indicate that *LRK2* can be a response to the plant peptide hormone PSK; The number of tillers, plant height, 1000-grain weight and number of grains per panicle of *LRK2* overexpressing plants at maturity were higher than those of wild type. The cold tolerance of *LRK2* overexpressing plants under cold stress was stronger than that of wild type. In addition, *LRK2* overexpressing plants could change the contents of malondialdehyde and proline in response to cold stress, which improved the cold tolerance of rice to some extent. The auxin response factor *OsARF24*, which interacts with *LRK2*, was screened by yeast two-hybrid and bimolecular fluorescence complementary experiments. For this purpose, we drew a model diagram of *LRK2* promoting tillering in rice and responding to PSK and low temperature stress (Figure 6).

## Figures and Tables

**Table 1:** Yield components in the wild-type and *LRK2* overexpressing plants in maturity.

Plant Line	Tiller Number per Plant	Plant Height (cm)	1000-Grain Weight (g)	Primary Branches	Secondary Branches	Panicle Length (cm)	Grains per Panicle
WT	11.33 ± 1.50	114.97± 1.12	25.24± 0.25	11.83 ±1.17	33.33 ± 2.73	22.45 ± 0.71	108 ± 5.12
OE-1	13.5 ± 1.05 *	112.22 ± 1.44	23.08± 0.10 **	12.10± 1.26	33.5 ± 2.17	23.67 ± 0.53 **	128 ± 3.04 *
OE-2	13.80 ± 1.32 *	112.07 ± 1.32	23.12 ± 0.3 **	12.56 ± 0.79	33.8 ± 2.30	23.78 ± 0.45 **	132 ± 2.56 *
OE-3	13.12± 1.47 *	113.03 ± 1.62	23.27 ± 0.06 **	12.83± 0.98	33.67 ± 2.16	23.93 ± 0.41 **	126 ± 1.51 *

n = 10, *t*-test, “*” is *p* < 0.05, “**” is *p* < 0.01.

## Data Availability

We obtained the OsLRK2, AtPSKR1, AtPSKR2, DcPSKR1 and SLPSKR1 protein sequences by using UniProt software (https://www.uniprot.org/, accessed on 15 December 2024). MEGA6.0 software was used to construct the phylogenetic tree. The conserved domain of protein was analyzed by Gendoc2.7. All other data supporting this result are included in the article.

## Supplementary Materials

The following supporting information can be downloaded at: https://www.mdpi.com/article/10.3390/plants13243569/s1, Figure S1. LRK2 and different species PSKR sequence analysis and LRK2 gene expression level analysis.

## References

[1] Zhu J.K. (2016). Abiotic Stress Signaling and Responses in Plants. Cell.

[2] Kovach M.J., Sweeney M.T., McCouch S.R. (2007). New insights into the history of rice domestication. Trends Genet..

[3] Saito K., Miura K., Nagano K., Hayano-Saito Y., Araki H., Kato A. (2001). Identification of two closely linked quantitative trait loci for cold tolerance on chromosome 4 of rice and their association with anther length. Theor. Appl. Genet..

[4] Ma Y., Dai X., Xu Y., Luo W., Zheng X., Zeng D., Pan Y., Lin X., Liu H., Zhang D. (2015). COLD1 confers chilling tolerance in rice. Cell.

[5] Zhang Z., Li J., Li F., Liu H., Yang W., Chong K., Xu Y. (2017). OsMAPK3 Phosphorylates OsbHLH002/OsICE1 and Inhibits Its Ubiquitination to Activate OsTPP1 and Enhances Rice Chilling Tolerance. Dev. Cell.

[6] Li R., Song Y., Wang X., Zheng C., Liu B., Zhang H., Ke J., Wu X., Wu L., Yang R. (2024). OsNAC5 orchestrates OsABI5 to fine-tune cold tolerance in rice. J. Integr. Plant Biol..

[7] Yang T., Shad Ali G., Yang L., Du L., Reddy A.S., Poovaiah B.W. (2010). Calcium/calmodulin-regulated receptor-like kinase CRLK1 interacts with MEKK1 in plants. Plant Signal Behav..

[8] Shiu S.H., Bleecker A.B. (2001). Receptor-like kinases from Arabidopsis form a monophyletic gene family related to animal receptor kinases. Proc. Natl. Acad. Sci. USA.

[9] Clark S.E., Williams R.W., Meyerowitz E.M. (1997). The CLAVATA1 gene encodes a putative receptor kinase that controls shoot and floral meristem size in Arabidopsis. Cell.

[10] Schoof H., Lenhard M., Haecker A., Mayer K.F., Jürgens G., Laux T. (2000). The stem cell population of Arabidopsis shoot meristems in maintained by a regulatory loop between the CLAVATA and WUSCHEL genes. Cell.

[11] Gómez-Gómez L., Boller T. (2000). FLS2: An LRR receptor-like kinase involved in the perception of the bacterial elicitor flagellin in Arabidopsis. Mol. Cell.

[12] Zipfel C., Kunze G., Chinchilla D., Caniard A., Jones J.D., Boller T., Felix G. (2006). Perception of the bacterial PAMP EF-Tu by the receptor EFR restricts Agrobacterium-mediated transformation. Cell.

[13] Gou X., He K., Yang H., Yuan T., Lin H., Clouse S.D., Li J. (2010). Genome-wide cloning and sequence analysis of leucine-rich repeat receptor-like protein kinase genes in Arabidopsis thaliana. BMC Genom..

[14] Walker J.C. (1994). Structure and function of the receptor-like protein kinases of higher plants. Plant Mol. Biol..

[15] Nagar P., Sharma N., Jain M., Sharma G., Prasad M., Mustafiz A. (2022). OsPSKR15, a phytosulfokine receptor from rice enhances abscisic acid response and drought stress tolerance. Physiol. Plant.

[16] Zhang H., Hu Z., Lei C., Zheng C., Wang J., Shao S., Li X., Xia X., Cai X., Zhou J. (2018). A Plant Phytosulfokine Peptide Initiates Auxin-Dependent Immunity through Cytosolic Ca^2+^ Signaling in Tomato. Plant Cell.

[17] Hartmann J., Fischer C., Dietrich P., Sauter M. (2014). Kinase activity and calmodulin binding are essential for growth signaling by the phytosulfokine receptor PSKR1. Plant J..

[18] Hartmann J., Stührwohldt N., Dahlke R.I., Sauter M. (2013). Phytosulfokine control of growth occurs in the epidermis, is likely to be non-cell autonomous and is dependent on brassinosteroids. Plant J..

[19] Li D., Sun C., Fu Y., Li C., Zhu Z., Chen L., Cai H., Wang X. (2002). Identification and mapping of genes for improving yield from Chinese common wild rice (*O. rufipogon* Griff.) using advanced backcross QTL analysis. Chin. Sci. Bull..

[20] Zha X., Luo X., Qian X., He G., Yang M., Li Y., Yang J. (2009). Over-expression of the rice *LRK1* gene improves quantitative yield components. Plant Biotechnol. J..

[21] Kang J., Li J., Gao S., Tian C., Zha X. (2017). Overexpression of the leucine-rich receptor-like kinase gene LRK2 increases drought tolerance and tiller number in rice. Plant Biotechnol. J..

[22] Wang Y., Xiong F., Nong S., Liao J., Xing A., Shen Q., Ma Y., Fang W., Zhu X. (2020). Effects of nitric oxide on the GABA, polyamines, and proline in tea (*Camellia sinensis*) roots under cold stress. Sci. Rep..

[23] Wang X., Zhuang N. (2008). Research progress of proline and cold resistance in plants. Chin. Agric. Sci. Bull..

[24] Li J., Geng G. (2003). Effects of low temperature stress on physiological and biochemical indexes of cold resistance of eggplant seedlings. J. Northwest A F Univ. (Nat. Sci. Ed.).

[25] Wu D., Mao D., Zhao X. (2022). Advances in molecular mechanisms of plant response to low temperature. Life Sci. Res..

[26] Velikova V., Yordanov I., Edreva A. (2000). Oxidative stress and some antioxidant systems in acid rain-treated bean plants. Plant Sci..

[27] Hao Y., Zhou M., Han K., Yang X., Gao Y., Chen Z. (2022). Cloning and expression analysis of three members of *PtYABBY*s in *Populus tomentosa*. Journal of Beijing Forestry University..

[28] Guilfoyle T.J., Hagen G. (2007). Auxin Response Factor. Curr. Opin. Plant Biol..

[29] Xu Q. (2023). Study on the Molecular Mechanism of the Regulation of Lip Development by PeARF18 Gene in *Phalaenopsis equestris*. Master’s Thesis.

[30] Okushima Y., Overvoorde P.J., Arima K., Alonso J.M., Chan A., Chang C., Ecker J.R., Hughes B., Lui A., Nguyen D. (2005). Functional genomic analysis of the AUXIN RESPONSE FACTOR gene family members in Arabidopsis thaliana: Unique and overlapping functions of ARF7 and ARF19. Plant Cell.

[31] Wang J.W., Wang L.J., Mao Y.B., Cai W.J., Xue H.W., Chen X.Y. (2005). Control of root cap formation by MicroRNA-targeted auxin response factors in Arabidopsis. Plant Cell.

[32] Wang B., Xue J.S., Yu Y.H., Liu S.Q., Zhang J.X., Yao X.Z., Liu Z.X., Xu X.F., Yang Z.N. (2017). Fine regulation of ARF17 for anther development and pollen formation. BMC Plant Biol..

[33] Chen J., Wang S., Wu F., Wei M., Li J., Yang F. (2022). Genome-Wide Identification and Functional Characterization of Auxin Response Factor (ARF) Genes in Eggplant. Int. J. Mol. Sci..

[34] Guan S.J., Wang N., Xu R.R., Ge T.T., Gao J., Yan Y.G., Zhang G., Chen Y., Zhang M.Y. (2021). Auxin Response Factor (ARF) Gene Family in *Glycyrrhiza uralensis* Fisch.: Identification and Expression Analysis. Chin. Agric. Sci. Bull..

[35] Chattopadhyay K., Chakraborty K., Samal P., Sarkar R.K. (2021). Identification of QTLs for stagnant flooding tolerance in rice employing genotyping by sequencing of a RIL population derived from *Swarna *× *Rashpanjor*. Physiol. Mol. Biol. Plants.

[36] Li Z., Fu D., Wang X., Zeng R., Zhang X., Tian J., Zhang S., Yang X., Tian F., Lai J. (2022). The transcription factor bZIP68 negatively regulates cold tolerance in maize. Plant Cell.

[37] Du H., Wang N.L., Xiong L.Z. (2018). Determination of Malondialdehyde (MDA) Content in Rice Leaves. Bio.

[38] Gehl C., Waadt R., Kudla J., Mendel R.R., Hänsch R. (2009). New GATEWAY vectors for high throughput analyses of protein-protein interactions by bimolecular fluorescence complementation. Mol. Plant.

[39] Tamura K., Stecher G., Peterson D., Filipski A., Kumar S. (2013). MEGA6: Molecular Evolutionary Genetics Analysis version 6.0. Mol. Biol. Evol..

