# Supplementary material for: A Leucine-Rich Receptor Like Kinase LRK2 Is Involved in the Regulation of Cold Tolerance and Yield in Rice
Source: Plants (Basel). 2024 Dec 21;13(24):3569. doi: 10.3390/plants13243569 (PMC11677858; doi:10.3390/plants13243569)
Supplement: Supplementary file 1 [file plants-13-03569-s001.zip › plants-3287479-supplementary.pdf]

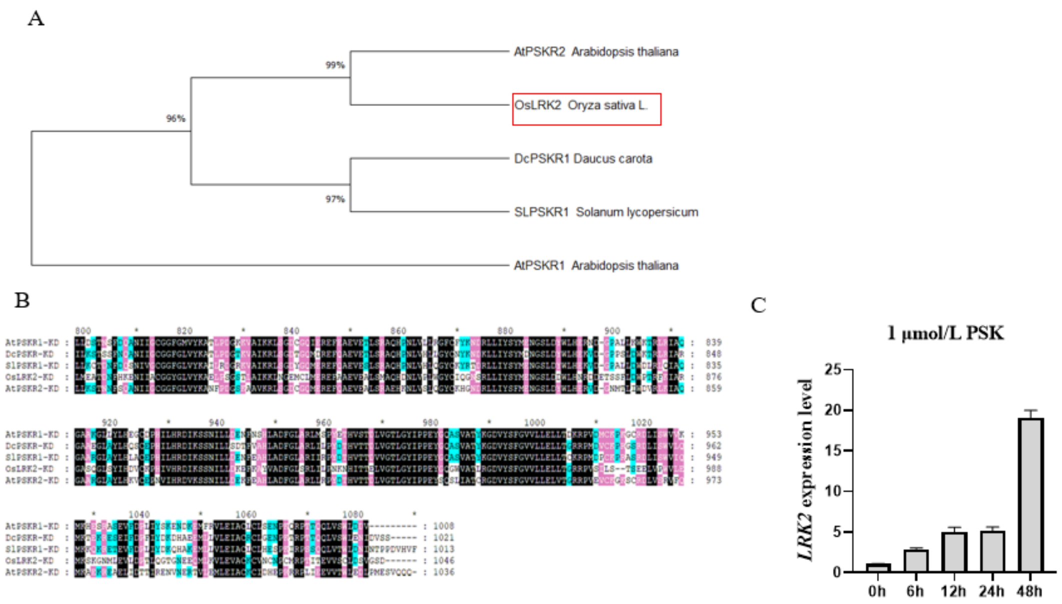

Figure S1. LRK2 and different species PSKR sequence analysis and *LRK2* gene expression level analysis. (A) Phylogenetic tree analysis of PSKR between LRK2 and different species. (B) Protein conserved domain analysis of LRK2 and PSKR in different species. (C) Expression of the *LRK2* gene in wide-type plants (*Oryza sativa L. ssp.* japonica cv. Zhonghua11) after treatment with 1 μmol/L PSK-*α*, analyzed using real-time quantitative PCR analysis (RT-qPCR).
